# Detection of Recurrent Cervical Cancer and Prediction of Its Patient Survival with Serum Squamous-Cell Carcinoma-Antigen and 2-[^18^F] Fluoro-2-Deoxy-d-Glucose-Positron Emission Tomography/Computed Tomography

**DOI:** 10.3390/diagnostics10090657

**Published:** 2020-08-31

**Authors:** Nan-Jing Peng, Chin Hu, Yu-Li Chiu, Chang-Ching Yu, Chia-Jung Li, Jim Jinn-Chyuan Sheu, An-Jen Chiang

**Affiliations:** 1 Department of Nuclear Medicine, Kaohsiung Veterans General Hospital, Kaohsiung 813, Taiwan; njpeng@vghks.gov.tw (N.-J.P.); ghu@vghks.gov.tw (C.H.); chiuyuli@vghks.gov.tw (Y.-L.C.); cccyu@vghks.gov.tw (C.-C.Y.); 2 School of Medicine, National Yang-Ming University, Taipei 112, Taiwan; 3 Department of Gynecology and Obstetrics, Kaohsiung Veterans General Hospital, Kaohsiung 813, Taiwan; nigel6761@gmail.com; 4 Institute of Biomedical Sciences, National Sun Yat-sen University, Kaohsiung 804, Taiwan; 5 Department of Biotechnology, Kaohsiung Medical University, Kaohsiung 807, Taiwan; 6 School of Chinese Medicine, China Medical University, Taichung 404, Taiwan; 7 Department of Health and Nutrition Biotechnology, Asia University, Taichung 413, Taiwan

**Keywords:** cervical cancer, FDG, imaging, metabolic tumor volume, PET/CT, SCC-Ag, squamous-cell carcinoma, total lesion glycolysis

## Abstract

Aim: To evaluate the usefulness of serum squamous-cell carcinoma antigen (SCC-Ag) and 2-[^18^F]fluoro-2-deoxy-D-glucose-positron emission tomography/computed tomography (FDG-PET/CT) for the detection of recurrent squamous-cell carcinoma (SqCC) of the uterine cervix, and its prediction of patient survival. Methods: FDG-PET/CT was performed for patients with serum SCC-Ag levels elevated to ≥1.5 ng/mL (Group 1) and those with suspicious recurrences without any increase in serum SCC-Ag levels (Group 2). The results were analyzed on the basis of histological data, disease progression and/or clinical follow-up. Recurrence was defined as evidence of recurrent lesions within 6 months of FDG-PET/CT. The outcome was determined using medical records. Results: In total, 88 consecutive patients with cervical SqCC cancer with suspected recurrence (62 in Group 1 and 26 in Group 2) were enrolled. Recurrences were observed in 55 patients (77.4% (48/62) in Group 1 vs. 26.9% (7/26) in Group 2, *p* < 0.001). The overall sensitivity, specificity and accuracy of serum SCC-Ag were 87.3%, 57.6% and 76.1%, respectively, and those of FDG-PET/CT were 98.2%, 90.9% and 95.5%, respectively; the corresponding values were 97.9%, 92.9% and 96.8% for Group 1 and 100%, 89.5% and 92.3% for Group 2. Surgical resection was performed for 16 patients. At the end of the study, 40.3% (25/62) of Group 1 patients and 88.5% (23/26) of Group 2 patients were alive (*p* < 0.001). The survival of patients who underwent surgical resection for recurrent tumors was higher than that of patients who did not undergo resection (62.5% (10/16) vs. 17.9% (7/39), *p* = 0.001). Metabolic tumor volume (MTV) and total lesion glycolysis (TLG) derived from FDG-PET/CT showed significantly different in-patient survival. Conclusions: Serum SCC-Ag could predict tumor recurrence and the survival of patients with SqCC cervical cancer. As such, the surgical resection of limited recurrent disease, as determined using FDG-PET/CT, might improve the survival of patients with cervical cancer. MTV and TLG may serve as a prognostic biomarker of survival in patients with recurrent cervical cancer.

## 1. Introduction

Uterine cervical cancer is the fourth most common cancer in women worldwide [1], but the incidence of cervical cancer has been reduced owing to economic development [2]. Accordingly, sub-Saharan Africa, South-East Asia, Latin America and the Caribbean, and Central and Eastern Europe have the highest rates of morbidity and mortality due to cervical cancer [3]. Twenty years ago, many Taiwanese women also had cervical cancer. According to the healthcare data from Taiwan, both the incidence and mortality rates of cervical cancer have decreased, although cervical cancer is the seventh most common cancer among women [4].

Considering the natural progression of cancer, the cure rate is high if the disease is detected early, but approximately one-third of patients treated for cervical cancer develop recurrences within the first 2 years of completing therapy [5]. If primary treatment fails, the secondary disease tends to exhibit even poorer cure rates [6]. Therefore, the early detection of cancer recurrence might influence survival [7]. Moreover, the detection of asymptomatic recurrences is associated with prolonged overall survival and survival from the time of the initial detection of recurrence [6]. Considering the patient benefits, attempts to improve surveillance after treatment might lead to the earlier detection of relapse, and precise assessment of the recurrence status could improve outcomes.

The SCC antigen is a tumor-associated protein of squamous-cell carcinoma of various organs. Increasing levels of tumor markers during follow-up can precede the clinical detection of tumor recurrence [8]. More than 85% of cervical cancers are of the squamous cell (SqCC) type. The serum squamous-cell carcinoma antigen (SCC-Ag) can indicate the active tumor status of patients with SqCC cervical cancer [9,10]. The increase in serum SCC-Ag levels above the identified thresholds in patients with recurrence was observed in 46% to 92% of patients, preceding a clinically apparent recurrence by an average of 2 to 8 months [11,12]. The use of this tumor marker has been shown to be cost-effective, but additional imaging examinations are needed to detect the exact tumor location and extension of recurrent lesions.

2-[^18^F] fluoro-2-deoxy-d-glucose positron emission tomography (FDG-PET) detects the increased glucose metabolism in malignant tumors. Combined PET and computed tomography (CT; PET/CT) has emerged as a promising imaging modality, and it is being applied more routinely in clinical situations. FDG-PET/CT has been increasingly recognized for its potential clinical utility in diagnosing patients with cervical cancer, and the prognostic value of PET/CT is associated with the presence and the level of FDG uptake [13,14,15,16,17]. FDG-PET/CT has the capacity to detect recurrent cervical cancer lesions, which precede the appearance of morphological changes detected via conventional imaging techniques [18,19]. The maximal standardized uptake value (SUVmax), which shows the highest uptake of a single tumor, is a widely used parameter in clinical practice. However, it could not represent the entire tumor activity, especially multiple lesions in the whole body. Recently, metabolic tumor volume (MTV) and total lesion glycolysis (TLG) have been adopted to measure the metabolic activity of the entire tumor burden in cervical cancer [20,21]. However, to our best knowledge, it is seldom used in the field of recurrent cervical cancer [22,23]. The early detection of recurrent disease improves the survival of patients with recurrent cervical cancer [7], particularly in patients who may benefit from surgery owing to the limited sites of metastatic disease [24,25]. MTV and TLG might better represent the prognostic value via the disease burden measures. Therefore, the current study aimed to evaluate the usefulness of serum SCC-Ag and FDG-PET/CT for the detection of recurrent SqCC cervical cancer and the prediction of patient survival.

## 2. Materials and Methods

### 2.1. Patients

We retrospectively collected the data of patients with prior cervical cancer after a complete response to treatment (primary surgery and/or chemoradiotherapy) who underwent FDG-PET/CT considering the following two points: (1) the decision to obtain a FDG-PET/CT scan was based on serum SCC-Ag levels ≥ 1.5 ng/mL; (2) FDG-PET/CT scans were obtained owing to suspected recurrence without any increase in the serum SCC-Ag level. The exclusion criteria of this study were as follows: (1) patients without available data about serum SCC-Ag; (2) patients receiving ongoing treatment and were not yet cured; (3) histological type other than SqCC; and (4) the presence of a second or third tumor. Between June 2006 and July 2018, 121 consecutive patients with prior cervical cancer underwent FDG-PET/CT. Of the patients, 33 were excluded: 19 patients with other histological types, 10 with double or triple cancers, 2 who were receiving ongoing treatment and were not cured, and 2 with no available data about serum SCC-Ag levels before FDG-PET/CT. Finally, 88 patients were recruited. In total, Sixty-two patients were referred owing to increasing serum SCC-Ag levels (Group 1), and the other 26 patients were referred because of suspected recurrence without any increase in serum SCC-Ag levels (Group 2). The characteristics of the enrolled patients are given in Table 1. According to the application form, the indications for suspected recurrence in Group 2 included suspicious or indeterminate findings on CT scans (*n* = 14), magnetic resonance imaging (*n* = 3) and chest film (*n* = 1), abdominal tenderness (*n* = 1), a palpable mass in the vagina (*n* = 2) and vaginal bleeding (*n* = 1), bone pain (*n* = 1), and elevation in the levels of other tumor markers (*n* = 3). The International Federation of Gynecology and Obstetrics (FIGO) classification was used for the initial staging of cervical cancer. This study was approved by the institutional review board and the hospital ethics committee (VGHKS18-CT1-04 (4 December 2017) and VGHKS19-CT12-03 (13 November 2019)). The need for written or verbal informed consent to participate in this study was waived by the hospital ethics committee because of the retrospective nature of the study.

### 2.2. Serum SCC-Ag Measurement

Peripheral blood from patients was obtained and centrifuged for 10 min at relative centrifugal force (RCF) 1300× *g*. Then serum was separated for examination. The serum SCC-Ag level was measured using a Chemiluminescent Microparticle Immunoassy (CMIA). The analysis was conducted with the patented products from Abbott Architect SCC, Tokyo, Japan, and the readout ensued automatically. The sensitivity of the Architect SCC assay was calculated to be better than 0.1 ng/mL. A level of serum SCC-Ag ≥ 1.5 ng/mL was regarded as elevated status.

### 2.3. FDG-PET/CT Imaging

The patients fasted for at least 6 h prior to FDG PET/CT imaging. An intravenous catheter was placed for the administration of radiopharmaceutical agents, and the blood glucose levels of the patients were measured prior to injection of the tracer. All of the patients had a blood glucose level of <150 mg/dL at the time of injection. Each patient received 370–555 MBq of ^18^F-FDG according to the patient’s body weight (7.03 MBq/kg). After injection of the tracer, the patients rested for 1 h on a comfortable bed in a dark room. Whole-body FDG-PET/CT (Discovery ST-16; GE Healthcare, Milwaukee, WI, USA) was performed from the head to the upper thigh while the patients were in a supine position. A delayed image with or without the use of diuretics was obtained when necessary. CT scanning was performed prior to acquisition of the PET data by using the following parameters: 0.6 s per rotation, 120 kV, 100 mA, and 3.75 mm thick slices. After the obtaining of a plain CT scan, PET images of the same areas were acquired in a two-dimensional mode; scans were collected every 4 min per bed position. Attenuation-corrected PET images were reconstructed by using an ordered subset expectation maximization iterative reconstructed algorithm. The 3.75 mm thick transaxial CT images were reconstructed at 3.27 mm intervals for fusion with the PET images. PET, CT and fused PET/CT images were generated on a Xeleris image display and processing platform (GE Healthcare) for review on a computer workstation.

### 2.4. Image Analysis

PET, CT and fused PET/CT images were interpreted by two qualified nuclear medicine physicians who were allowed to manipulate the image contrast, image intensity and three-dimensional images on a computer screen. The final diagnosis was made via consensus. The physicians were not blinded to the reason for examination and the outcomes at the time of image analysis. As this was a retrospective study, the reasons for examination were described on the application form. Each study was considered an independent event, and data about imaging modalities including prior PET/CT and contrast-enhanced CT were available at the time of review for the best reading. Both the physicians independently reviewed the scans before arriving at a consensus. Any increase in the uptake of FDG was compared with the corresponding anatomical finding on the CT scan. The areas of increased FDG uptake that corresponded to normal structures such as the muscle and fat tissues or the bowel were considered to indicate physiologic uptake or benign processes. For lesions that demonstrated abnormal FDG uptake, the physician outlined the region of interest as the area with the greatest amount of uptake. The standardized uptake value (SUV) was determined semi-automatically by using the SUV tools available in the Xeleris software as follows: SUV = activity in the region of interest (Bq/g)/(injected dose (Bq)/body weight (g)). The two-dimensional regions of interest were drawn around the tumor on each transaxial slice that contained tumor tissue. The single-pixel maximal SUV (SUVmax) was determined for each region, and the slice that demonstrated the highest SUV was considered the SUVmax for the entire tumor.

MTV refers to the volume of tumor that has SUV over a certain threshold of SUV. TLG was calculated by multiplying the SUV of the tumors by the MTV. Using the PET VCAR (volume computer assisted reading) application, we drew a cuboid volume of interest (VOI) covering a cancer lesion and then VOI was automatically drawn along the margin of the tumor uptake according to the specific SUV threshold. MTV and TLG were automatically calculated by PET VCAR application (Advanced workstation 4.4, GE Medical System, Milwaukee, WI, USA).

### 2.5. Statistical Analysis

The results regarding disease progression were analyzed via pathological examination when histological sampling was possible and/or during clinical follow-up. Recurrent cervical cancer was defined as the detection of recurrent lesions within 6 months of obtaining the FDG-PET/CT scan. The chi-square test was used to determine the significance of the differences between the two groups considering the detection of recurrent disease and their outcomes. The differences were considered significant at *p* < 0.05. A cumulative survival curve was derived using the Kaplan–Meier method. All the calculations were performed using SPSS software, version 20.0 (SPSS, Chicago, IL, USA).

## 3. Results

The outcomes of FDG-PET/CT scans in Group 1 and Group 2 patients are listed in Table 2. Among the 88 patients who underwent FDG-PET/CT scans, the mean SCC-Ag level at the time of FDG-PET/CT imaging was 10.3 ng/mL (range, 1.5–64.2 ng/mL) for Group 1 and 0.8 ng/mL (range, 0.1–1.4 ng/mL) for Group 2 (*p* = 0.004). There were no significant differences in the time from diagnosis of cancer, mean follow-up time and time to recurrence. Recurrences were confirmed in 55 patients, including 48 of 62 (77.4%) in Group 1 and 7 of 26 (26.9%) in Group 2 (*p* < 0.001). In total, 29 of the patients (52.7%) were proven to have recurrence using histology, while the others were confirmed via the findings of disease progression or their response to therapy upon follow-up. Surgical resection was performed in 27.1% (13/48) of patients with recurrence in Group 1 and 42.9% (3/7) in Group 2. At the end of the study, 37 of the 62 (59.7%) patients in Group 1 and 3 of the 22 (11.5%) patients in Group 2 were dead (*p* < 0.001).

The use of FDG-PET/CT resulted in the detection of recurrent cancer in 54 patients. False-positive results were also identified in 3 patients. Two of the patients were proven to have second primary cancers of urothelial cell carcinoma and multiple myeloma, respectively, on histology, and the remaining had an abscess over the rectovaginal fistula. A false-negative result was noted in Group 1; the patient had metastatic nodes in the abdomen, which were masked owing to urinary stasis. In the remaining patients, no recurrent disease was detected during a follow-up of at least 6 months including clinical surveillance, serum SCC-Ag level evaluation and other imaging modalities. In Group 1, FDG-PET/CT revealed recurrent cancers in 47 patients, a false-positive result in 1, and a false-negative result in 1. In Group 2, FDG-PET/CT revealed recurrent cancers in seven patients and false-positive results in two. Accordingly, FDG-PET/CT had the sensitivity, specificity and accuracy of 98.2%, 90.9% and 95.5%, respectively, with corresponding values of 97.9%, 92.9% and 96.8% for Group 1 and 100%, 89.5% and 92.3% for Group 2; in contrast, the sensitivity, specificity, and accuracy of serum SCC-Ag level measurements were 87.3%, 57.6% and 76.1%, respectively (Table 3).

**Figure 1:**
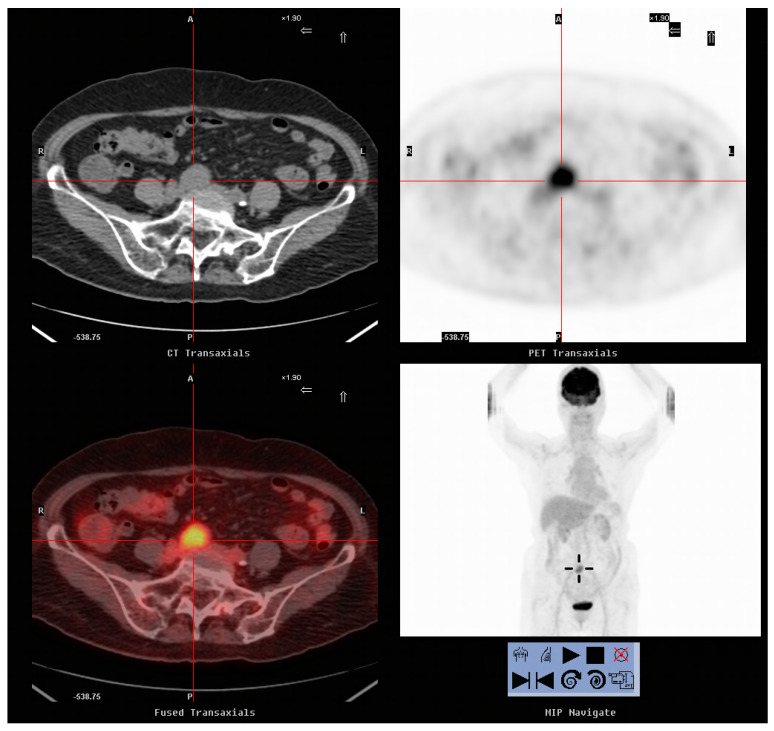
A 78-year-old woman who had cervical cancer (SqCC, carcinoma in situ) 10 years previously presented with the elevation of the serum SCC-Ag level to 6.7 ng/mL. FDG-PET/CT revealed a 3.0 cm FDG-avid nodule over the right pre-sacral area (SUVmax: 5.2, cross-cursor), indicating a recurrent lesion. The patient underwent resection of the nodule, and pathology indicated a metastatic node. Currently, the patient has been disease-free for more than 10 years.

**Figure 2:**
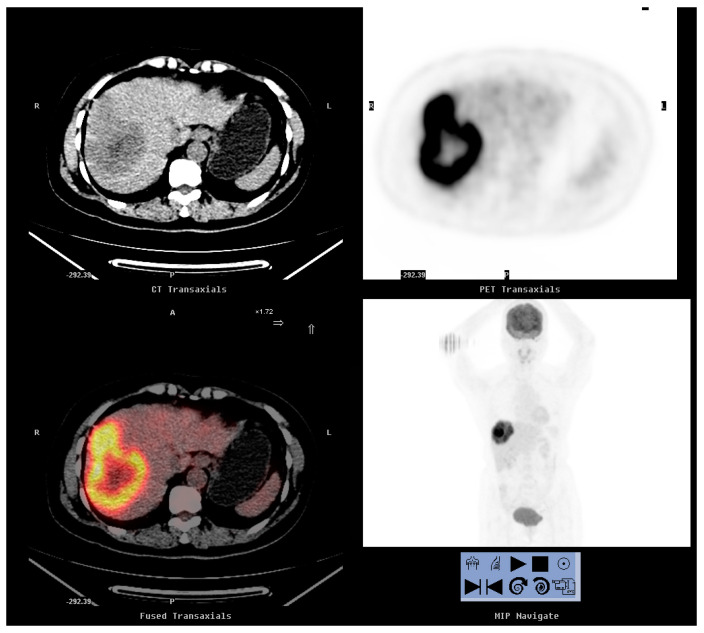
A 56-year-old woman who had cervical cancer (SqCC, FIGO stage IB1) 5 years previously presented with an elevation of the serum SCC-Ag level to 1.8 ng/mL. FDG-PET/CT revealed an 11.0 cm FDG-avid hypodense nodule in the right hepatic lobe (SUVmax: 7.9). The patient underwent right lobectomy, and pathology revealed a moderately differentiated metastatic SqCC, compatible with the findings of a tumor with cervical origin. However, the tumor relapsed, and her disease progressed. The patient died 3 years after the FDG-PET/CT examination.

As shown in Table 4, 18 patients underwent surgical resection of the tumors. Of these, 16 were proven to have recurrent cervical cancer upon pathological examination (Figure 1 and Figure 2). The remaining two were proven to have a second primary cancer and a rectovaginal abscess, respectively. On comparing patients with recurrence treated with surgical resection and those who did not undergo resection, the mean SCC-Ag level at the time of FDG-PET/CT imaging, mean SUVmax and time to recurrence were not significantly different (Table 5). The mean follow-up times were 56.06 months and 21.18 months, respectively (*p* = 0.002). Cervical cancer was confirmed on histology for all the 16 patients (100%) who underwent surgical resection and 13 of 39 patients (33.3%) who did not undergo resection (*p* < 0.001). At the end of the study, 6 of the 16 patients (37.5%) who underwent surgical resection for recurrent lesions and 32 of the 39 (82.1%) who did not were dead (*p* = 0.001). The times from recurrence to death were 28.2 months (range, 10–37 months) and 12.3 months (range, 2–32 months), respectively (*p* = 0.015).

**Figure 3:**
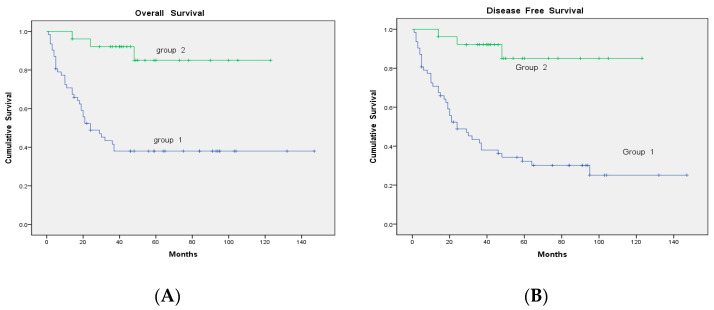
Comparison of survival outcomes: (**A**) overall survival (OS) and (**B**) disease-free survival (DFS) between Group 1 and Group 2 patients.

**Figure 4:**
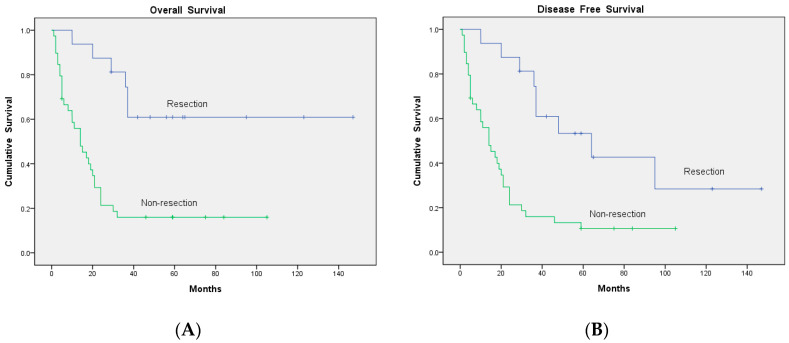
Comparison of survival outcomes: (**A**) overall survival (OS) and (**B**) disease-free survival (DFS) according to whether surgical resection was performed for the tumor lesions.

After continuous follow-up, nine patients in Group 1 developed recurrence again. Five of them were still alive at the end of the study. The overall survival (OS) and disease-free survival (DFS) were higher in Group 2 than in Group 1 (25/62 vs. 23/26, *p* < 0.001; and 20/62 vs. 23/26, *p* < 0.001, respectively; Figure 3). Three of the 16 patients who underwent surgical resection for tumors developed recurrence again, whereas 2 of the 39 patients who did not undergo resection showed recurrence. The OS and DFS were higher in patients who underwent surgical resection than in those who did not (10/16 vs. 7/39, *p* = 0.001; and 7/16 vs. 5/39, *p* = 0.012, respectively; Figure 4).

This section may be divided by subheadings. It should provide a concise and precise description of the experimental results, their interpretation as well as the experimental conclusions that can be drawn.

There is significant difference in OS and DFS between patients with positive PET/CT and those with negative PET/CT (20/57 vs. 28/31, *p* < 0.001; and 15/57 vs. 28/31, *p* < 0.001, respectively). For the 54 recurrent patients with positive FDG-PET/CT, the values of SCC-Ag, tumor SUVmax, MTV and TLG are listed in Table 6. The cut-off values for predicting prognosis were determined using the values of variables above the median. Significant differences were observed in the OS of SUVmax, MTV and TLG (13/27 vs. 4/27, *p* = 0.018; 14/27 vs. 3/27, *p* = 0.003; and 16/27 vs. 1/27, *p* < 0.001, respectively) and the DFS of MTV and TLG (11/27 vs. 1/27, *p* = 0.002; and 11/27 vs. 1/27, *p* = 0.002, respectively).

## 4. Discussion

The measurement of the serum SCC-Ag level is helpful for monitoring the therapeutic response of cervical SqCC and for the surveillance of recurrent disease [26]. However, increased serum SCC-Ag levels are also observed in SqCC of the esophagus, lungs, and head and neck, as well as in some benign diseases such as skin disorders, pelvic inflammatory disease, cystitis and renal failure [8,27,28]. Oh et al. conducted a study to determine the optimal cutoff level of serum SCC-Ag to detect recurrent cervical SqCC during post-treatment surveillance [26]. The sensitivity was 92.5%, 86.3%, 80.2%, 73.1% and 67.8%, while the specificity was 50.3%, 85.0%, 94.6%, 97.7% and 98.9% when the threshold settings were identified to be ≥1.0, ≥1.5, ≥2.0, ≥2.5 and ≥3.0 ng/mL, respectively. They concluded that a higher cutoff level would have a lower sensitivity but higher specificity, and vice versa. In the present study, the measurement of the serum SCC-Ag level had a sensitivity and specificity of 87.3% and 57.6%, respectively, for the detection of recurrent cervical SqCC when the threshold setting was ≥ 1.5 ng/mL, which were within the reported ranges of 86.3–100% and 47.4–85.0%, respectively [26,29,30]. Patients with recurrent tumors generally have a poor prognosis. Many factors would affect the prognoses of patients with recurrence, including the initial clinical stage, presence of lymphatic metastasis, site of recurrence, level of SCC-Ag prior to recurrence, and change in SCC-Ag levels during treatment [10,31]. Wang et al. reported that 161 of 180 (89.4%) patients with recurrences had elevated serum SCC-Ag levels [10]. The clinical stage, site of recurrence and serum SCC-Ag levels after treatment were closely associated with the survival time of the patients. In the present study, we observed that the incidences of recurrence and mortality were higher in patients with elevated serum SCC-Ag levels than in those without (77.4% (48/62) vs. 26.9% (7/26), *p* < 0.001; and 59.7% (37/62) vs. 11.5% (3/26), *p* < 0.001, respectively). Meanwhile, the OS and DFS were significantly lower in patients with elevated SCC-Ag levels than in those without (*p* < 0.001 and *p* < 0.001, respectively), indicating that patients with elevated SCC-Ag levels have a low survival rate. The prognostic role of SCC-Ag in recurrent cervical cancer is consistent with the meta-analysis results of a recently published article [32].

FDG-PET/CT is useful for the detection of recurrent cervical cancer. The reported sensitivity and specificity were 93–100% and 59–89%, respectively [33]. In the present study, FDG-PET/CT detected recurrent cervical cancer in 54 of 55 patients. The sensitivity, specificity and accuracy of FDG-PET/CT for recurrent cancer were 98.2%, 90.9% and 92.5%, respectively. No significant differences were observed considering the sensitivity between FDG-PET/CT and serum SCC-Ag levels, but FDG-PET/CT had a higher specificity and accuracy than serum SCC-Ag levels (90.9% vs. 57.6%, *p* < 0.001; and 95.5% vs. 76.1%, *p* = 0.004, respectively). Zhou et al. conducted a retrospective review of 17 studies with 707 patients who underwent FDG-PET/CT scans for the detection of the recurrence of cervical cancer; the sensitivity and specificity of FDG-PET/CT were 97% (range, 95–99%) and 81% (range, 74–87%), respectively. In contrast, the sensitivity and specificity owing to elevated serum SCC-Ag levels were 99% (range, 93–100%) and 77% (range, 59–89%), respectively [32]. They suggested that FDG-PET/CT is a more suitable option for detecting suspected cases of recurrent cervical cancer, especially in patients who have elevated serum SCC-Ag levels. Sookha et al. showed that the sensitivity, specificity and accuracy of FDG-PET/CT for detecting recurrent cervical cancer were 100%, 70.6% and 88.1%, respectively; the accuracy of using combined elevated SCC-Ag and carcinoembryonic antigen (CEA) levels was 100% compared to 90.0% when using only elevated SCC-Ag levels, and 33.3% when only elevated CEA levels, were used (*p* = 0.036) [29]. However, the numbers of cases evaluated by using only elevated SCC-Ag levels and only elevated CEA levels were small (9/10 vs. 2/6). In the current study, no significant differences were observed in the sensitivity, specificity and accuracy for patients with elevated serum SCC-Ag levels (97.9%, 92.9% and 96.8%, respectively) or for those without any elevated levels (100%, 89.5% and 92.3%, respectively). Therefore, elevated serum SCC-Ag levels may only indicate recurrence, with the higher probability of FDG-PET/CT detecting recurrent cervical cancer (77.4% (48/62) vs. 26.9% (7/26), *p* < 0.001). The results of the current study confirmed that FDG-PET/CT is valuable for detecting suspected recurrent cervical cancer, regardless of whether patients showed elevation of serum SCC-Ag levels.

Surgery has curative potential for patients with limited sites of metastatic disease. Several studies showed a survival benefit of surgical resection for pelvic recurrence, isolated para-aortic lymph node recurrence and pulmonary metastasis [24,25,33,34] However, simply monitoring the serum SCC-Ag levels might not be sufficient for the early detection of recurrence, especially for resectable disease. FDG-PET/CT has the advantages of merging the metabolic information obtained via FDG-PET with the anatomical information obtained via CT. In the present study, among patients who underwent surgical resection and in whom recurrent disease was detected early, 6 of the 16 (37.5%) patients who underwent surgical resection for the recurrent lesion and 32 of the 39 (82.1%) patients who did not undergo resection died at the end of the study (*p* = 0.001). The earlier recognition of resectable lesions should help in providing a greater number of effective treatment options, thereby improving survival. Accordingly, FDG-PET/CT is helpful for selecting patients who may derive a significant survival benefit from the performance of an optimal surgical strategy. Therefore, the OS and DFS were higher in patients who underwent surgical resection than in those who did not (*p* = 0.001 and *p* = 0.012, respectively).

Volume-based FDG-PET/CT parameters have been adopted to measure the metabolic activity of the entirety of the tumors in the whole body. MTV and TLG determined by FDG-PET/CT have the potential to improve the value of SUVmax and enhance the prognostic value of disease burden measures. Sun et al. reviewed 91 consecutive cervical cancer patients’ whole body FDG-PET/CT images and measured their volume-metabolic parameters. The OS was 88.8% for patients with low MTV and 45.5% for those with high MTV. Univariate analysis showed that MTV and TLG were significant prognostic factors for OS. Upon multivariate analysis, MTV remained significant for OS [20]. Han et al. conducted a systematic review and meta-analysis study including 660 cervical cancer patients from 12 studies. They found that prognoses were worse in patients with high MTV and TLG, with pooled hazard ratios of 5.89 and 5.82, respectively [21]. A study of 22 post-irradiated recurrent cervical cancer patients showed that one year DFS was 28% for patients with SUVmax values of >5.8 versus 42% for those with SUVmax values of <5.8 (*p* = 0.01), but 43% for patients with MTV values of >43 cm^3^ versus 45% for those with MTV values of <43 cm^3^ (*p* = 0.8) [22]. Another study of 26 patients with recurrent cervical cancer demonstrated that SUVmax and distant metastasis were independent predictors of OS and DFS on multivariate analysis. MTV, TLG and regional node involvement were found to be significant on univariate analysis, but not on multivariate analysis [23]. In this study with recurrent cervical cancer, we found significantly higher OS and DFS in patients with lower MTV and TLG, and vice versa. Our data for recurrent cervical cancer are in agreement with a number of studies observed in cervical cancers [20,21,35,36,37], and suggest that MTV and TLG is a prognostic biomarker of survival in patients with recurrent cervical cancer. Higher MTV or TLG carried a higher risk of adverse events or death.

FDG-PET/CT has some inherent false-positive and false-negative results. However, patients might also benefit from these false-positive findings. In the present study, false-positive results were the reason why surgical resection was performed for a second primary cancer of urothelial cell carcinoma and further treatment was performed for a vaginorectal abscess. The earlier recognition of synchronous cancer and infectious focus might allow the performance of more effective treatment options and improve patient survival. A false-negative result that was derived from the definition of recurrent lesions was detected within 6 months of FDG-PET/CT. FDG-PET/CT was limited by the resolution and the difficulty of detecting some metastases. The possible causes for false-negative results on FDG-PET were as follows: (1) cancers with hypometabolic features or low accumulations of FDG; (2) cancers with low cell density; (3) cancers with high background activity (e.g., urinary tract cancers); (4) and small cancers [38,39,40].

The present study had some limitations. First, pathology data were available for 37 of 88 (42.0%) patients. Recurrent tumors were confirmed in 29 of 55 (52.7%) patients: recurrent tumors were identified in 19 patients considering the disease progression and in another 7 patients on the basis of their response to therapy on follow-up. Second, the current study was a retrospective study, and 33 of 121 (27.3%) patients were not included in the analysis according to the exclusion criteria. Furthermore, FDG-PET/CT in Group 2 patients was performed owing to various reasons. As physicians collected the available information, including data regarding prior PET/CT and contrast-enhanced CT, bias might have occurred. Third, the total number of cases was small. Hence, further studies that include greater numbers of cases and prospective trials that focus on the specific issue of resectable recurrence are required.

## 5. Conclusions

The recurrence of cervical cancer has a significant impact on prognosis, and the early detection of disease recurrence might improve the OS and DFS of these patients. Serum SCC-Ag can serve as a surrogate as a deciding metric for the recurrence of cervical SqCC that is just as effective as FDG-PET/CT. FDG-PET/CT had a high sensitivity and specificity for detecting recurrent cervical SqCC, both in patients with and without elevated serum SCC-Ag levels. FDG-PET/CT enabled the early detection of resectable recurrence, thereby assisting physicians in changing the planned management. The surgical resection of limited recurrent disease according to the FDG-PET/CT data improved the survival of patients with recurrent cervical SqCC. MTV and TLG derived from FDG-PET/CT were associated with high-risk features and may serve as a prognostic biomarker of survival in patients with recurrent cervical cancer. Therefore, we recommend that FDG-PET/CT should be performed for not only patients with elevated serum SCC-Ag levels, but also those with suspected recurrence, as it will help in the earlier detection of resectable disease and predict the prognosis.

## Figures and Tables

**Table 1:** Characteristics of the enrolled Group 1 and Group 2 patients.

		Group 1 (*n* = 62)	Group 2 (*n* = 26)	*p*
Age		57.9 ± 10.53	55.54 ± 8.78	0.283
Stage	FIGO			
CIS		4 (6.5)	2 (7.7)	1.000
IA		5 (8.1)	0 (0)	0.316
IB		18 (29)	6 (23.1)	0.567
IIA		2 (3.2)	1 (3.8)	1.000
IIB		10 (16.1)	4 (15.4)	1.000
IIIA		0 (0)	1 (3.8)	0.295
IIIB		10 (16.1)	9 (34.6)	0.054
IVA		2 (3.2)	0 (0)	1.000
IVB		8 (12.9)	1 (3.8)	0.271
Unknown		3 (4.8)	2 (7.7)	0.630
Treatment				
OP		8 (12.9)	6 (23.1)	0.234
C/T		4 (6.5)	0 (0)	0.315
R/T		4 (6.5)	1 (3.8)	1.000
CCRT		11 (17.7)	13 (50)	0.002
OP + C/T		6 (9.7)	0 (0)	0.174
OP + R/T		9 (14.5)	3 (11.5)	1.000
OP + CCRT		15 (24.2)	2 (7.7)	0.084
OP + C/T + R/T		5 (8.1)	1 (3.8)	0.665

CCRT: concurrent chemoradiotherapy; CIS: carcinoma in situ; C/T: chemotherapy; OP: operation; R/T: radiotherapy.

**Table 2:** Outcomes of FDG-PET/CT scans in Group 1 and Group 2 patients.

	Group 1 (*n* = 62)	Group 2 (*n* = 26)	*p*
Time from diagnosis of cancer, months (range)	61.39 (4–432)	64.69 (4–360)	0.894
Mean SCC-Ag level, ng/mL (range)	10.3 (1.5–64.2)	0.8 (0.1–1.4)	0.004
Mean follow-up time, months (range)	37.74 (1–147)	53.08 (14–123)	0.059
Recurrence, N (%) *	48 (77.4%)	7 (26.9%)	<0.001
Time to recurrence, months (range)	50.65 (5–432)	98.86 (7–360)	0.517
Histology, N (%) *	26 (54.2%)	3 (42.9%)	0.696
Surgical resection, N (%) *	13 (27.1%)	3 (42.9%)	0.402
Death, N (%) *	37 (59.7%)	3 (11.5%)	<0.001

*: Patients with recurrence; FDG-PET/CT, 2-[^18^F]fluoro-2-deoxy-D-glucose-positron emission tomography/computed tomography; SCC-Ag, squamous-cell carcinoma antigen.

**Table 3:** Effectiveness of serum SCC-Ag levels and FDG-PET/CT for detecting recurrent cancer.

	TP	FP	TN	FN	Sensitivity	Specificity	Accuracy
Serum SCC-Ag (*n* = 88)	48	14	19	7	87.3%	57.6% *	76.1% ^†^
FDG-PET/CT (*n* = 88)	54	3	30	1	98.2%	90.9% *	95.5% ^†^
Group 1 FDG-PET/CT (*n* = 62)	47	1	13	1	97.9%	92.9%	96.8%
Group 2 FDG-PET/CT (*n* = 26)	7	2	17	0	100%	89.5%	92.3%

* *p* < 0.001 FDG-PET/CT vs. serum SCC-Ag; ^†^ *p* = 0.004 FDG-PET/CT vs. serum SCC-Ag; FDG-PET/CT, 2-[^18^F] fluoro-2-deoxy-D-glucose-positron emission tomography/computed tomography; FN, false negative; FP, false positive; SCC-Ag, squamous-cell carcinoma antigen; TN, true negative; TP, true positive.

**Table 4:** FDG-PET/CT findings and management of the 18 patients who underwent surgical resection.

Case	Age (Year)	SCC-Ag (ng/mL)	Stage	FDG-PET/CT Findings	SUVmax	Pathology	Follow-Up Time (Month)
1	78	6.7	0	A 3 cm nodule over right pre-sacral area	5.2	SqCC	147
2	58	1.9	IA	A 2.6 cm pelvic nodule	7.1	SqCC	20
3	68	50.3	IVA	Pelvic masses	11.2	SqCC	10
4	57	1.8	0	A 2 cm nodule in vaginal stump	8.1	SqCC	95
5	56	3.9	IVA	A 3.2 cm nodule at posterior pelvic region	12.7	SqCC	37
6	42	4.4	IB	RML nodule	13.8	SqCC *	36
7	48	2.8	IB1	Bilateral inguinal nodes	8.4	SqCC	65
8	61	3.7	IIA1	Left para-aortic and inguinal nodes	6.7	SqCC	64
9	56	9.5	0	Vaginal cuff and left pelvic nodes	4.9	SqCC	48
10	55	2.2	0	A 3.3 cm LUL nodule	16.9	SqCC *	56
11	45	2.8	0	A 3.3 cm left pelvic nodule	9.8	SqCC	37
12	56	1.8	IB1	A 11 cm mass in liver	22.2	SqCC *	29
13	49	11.4	IVB	A 4.1 cm mass in lower vagina to vulva	11.1	SqCC	30
14	56	0.7	II	Uterine cervix	3.1	SqCC	29
15	49	0.9	IB1	RUL and RLL nodules	4.2	SqCC *	42
16	46	0.1	IB	A 1.3 cm left para-aortic nodule	5.3	SqCC	123
17	69	28	IIA	A 6 cm mass involving lower right kidney and psoas muscle	16.4	UCC	24
18	45	1.0	IIB	A 1.5 cm nodule in cervix uteri	7.2	Abscess	73

*, origin in the cervix and uterus; FDG-PET/CT, 2-[^18^F]fluoro-2-deoxy-D-glucose-positron emission tomography/computed tomography; LUL, left upper lobe of lungs; RLL, right lower lobe of the lungs; RML, right middle lobe of the lungs; RUL, right upper lobe of the lungs; SCC-Ag, squamous-cell carcinoma antigen; SqCC, squamous-cell carcinoma; SUVmax, maximal standardized uptake; UCC, urothelial cell carcinoma.

**Table 5:** Outcomes of patients with recurrence treated with or without surgical resection.

	Surgical Resection (*n* = 16)	Non-Surgical Resection (*n* = 39)	*p*
Mean SCC-Ag level, ng/mL (range)	6.63 (0.7–50.3)	12 (0.4–70)	0.208
Mean SUVmax (range)	9.88 (3.1–22.2)	11.85 (4.8–60.4)	0.298
Time to recurrence, months (range)	71.63 (8–360)	50.69 (5–432)	0.416
Mean follow-up time, months (range)	56.06 (10–147)	21.18 (1–105)	0.002
Histology, N (%) *	16 (100%)	13 (33.3%)	<0.001
Death, N (%) *	6 (37.5%)	32 (82.1%)	0.001
Time from recurrence to death, months (range)	28.2 (10–37)	12.3 (2–32)	0.015

*: Patients with recurrence; SCC-Ag, squamous-cell carcinoma antigen; SUVmax, maximal standardized uptake.

**Table 6:** The survival outcomes of 54 recurrent patients with positive FDG-PET/CT according to the values of SCC, SUVmax, MTV and TLG.

	Madian (Range)	Cutoff	OS (%)	*p*	DFS (%)	*p*
SCC-Ag (ng/mL)	4.2 (0.1–70)	≤4.2	11 (40.7)	0.143	8 (29.6)	0.327
		>4.2	6 (22.2)		4 (14.8)	
SUVmax	10.1 (3.1–60.4)	≤10.1	13 (48.1)	0.018	9 (33.3)	0.099
		>10.1	4 (14.8)		3 (11.1)	
MTV(cm^3^)	30 (1–1493.0)	≤30	14 (51.9)	0.003	11 (40.7)	0.002
		>30	3 (11.1)		1 (3.7)	
TLG(mlxcm^3^)	139.6 (4.6–4196.5)	≤139.6	16 (59.3)	<0.001	11 (40.7)	0.002
		>139.6	1 (3.7)		1 (3.7)	

DFS, disease-free survival; MTV, metabolic tumor volume; OS, overall survival; SCC-Ag, squamous-cell carcinoma antigen; SUVmax, maximal standardized uptake; TLG, total lesion glycolysis.

## References

[1] Torre L.A., Bray F., Siegel R.L., Ferlay J., Lortet-Tieulent J., Jemal A. (2015). Global cancer statistics, 2012. CA Cancer J. Clin..

[2] Jemal A., Bray F., Center M.M., Ferlay J., Ward E., Forman D. (2011). Global cancer statistics. CA Cancer J. Clin..

[3] Arbyn M., Weiderpass E., Bruni L., de Sanjose S., Saraiya M., Ferlay J. (2020). Estimates of incidence and mortality of cervical cancer in 2018: A worldwide analysis. Lancet Glob. Health.

[4] Shen S.C., Hung Y.C., Kung P.T., Yang W.H., Wang Y.H., Tsai W.C. (2016). Factors involved in the delay of treatment initiation for cervical cancer patients: A nationwide population-based study. Medicine (Baltimore).

[5] Waggoner S.E. (2003). Cervical cancer. Lancet.

[6] Bodurka-Bevers D., Morris M., Eifel P.J., Levenback C., Bevers M.W., Lucas K.R., Wharton J.T. (2000). Posttherapy surveillance of women with cervical cancer: An outcomes analysis. Gynecol. Oncol..

[7] Sakurai H., Suzuki Y., Nonaka T., Ishikawa H., Shioya M., Kiyohara H., Katoh H., Nakayama Y., Hasegawa M., Nakano T. (2006). Fdg-pet in the detection of recurrence of uterine cervical carcinoma following radiation therapy—Tumor volume and fdg uptake value. Gynecol. Oncol..

[8] Gadducci A., Tana R., Cosio S., Genazzani A.R. (2008). The serum assay of tumour markers in the prognostic evaluation, treatment monitoring and follow-up of patients with cervical cancer: A review of the literature. Crit. Rev. Oncol. Hematol..

[9] Reesink-Peters N., van der Velden J., Ten Hoor K.A., Boezen H.M., de Vries E.G., Schilthuis M.S., Mourits M.J., Nijman H.W., Aalders J.G., Hollema H. (2005). Preoperative serum squamous cell carcinoma antigen levels in clinical decision making for patients with early-stage cervical cancer. J. Clin. Oncol..

[10] Wang Y., Cui T., Du L., Xu X., Tian B., Sun T., Han C., Zhao X., Jing J. (2017). The correlation between the serum squamous carcinoma antigen and the prognosis of recurrent cervical squamous carcinoma. J. Clin. Lab. Anal..

[11] Bolli J.A., Doering D.L., Bosscher J.R., Day T.G., Rao C.V., Owens K., Kelly B., Goldsmith J. (1994). Squamous cell carcinoma antigen: Clinical utility in squamous cell carcinoma of the uterine cervix. Gynecol. Oncol..

[12] Esajas M.D., Duk J.M., de Bruijn H.W., Aalders J.G., Willemse P.H., Sluiter W., Pras B., ten Hoor K., Hollema H., van der Zee A.G. (2001). Clinical value of routine serum squamous cell carcinoma antigen in follow-up of patients with early-stage cervical cancer. J. Clin. Oncol..

[13] Belhocine T. (2003). An appraisal of 18f-fdg pet imaging in post-therapy surveillance of uterine cancers: Clinical evidence and a research proposal. Int. J. Gynecol. Cancer.

[14] Yen T.C., Ng K.K., Ma S.Y., Chou H.H., Tsai C.S., Hsueh S., Chang T.C., Hong J.H., See L.C., Lin W.J. (2003). Value of dual-phase 2-fluoro-2-deoxy-d-glucose positron emission tomography in cervical cancer. J. Clin. Oncol..

[15] Grigsby P.W., Siegel B.A., Dehdashti F. (2001). Lymph node staging by positron emission tomography in patients with carcinoma of the cervix. J. Clin. Oncol..

[16] Kidd E.A., Siegel B.A., Dehdashti F., Grigsby P.W. (2007). The standardized uptake value for f-18 fluorodeoxyglucose is a sensitive predictive biomarker for cervical cancer treatment response and survival. Cancer.

[17] Nakamura K., Okumura Y., Kodama J., Hongo A., Kanazawa S., Hiramatsu Y. (2010). The predictive value of measurement of suvmax and scc-antigen in patients with pretreatment of primary squamous cell carcinoma of cervix. Gynecol. Oncol..

[18] Yen T.C., See L.C., Chang T.C., Huang K.G., Ng K.K., Tang S.G., Chang Y.C., Hsueh S., Tsai C.S., Hong J.H. (2004). Defining the priority of using 18f-fdg pet for recurrent cervical cancer. J. Nucl. Med..

[19] Havrilesky L.J., Wong T.Z., Secord A.A., Berchuck A., Clarke-Pearson D.L., Jones E.L. (2003). The role of pet scanning in the detection of recurrent cervical cancer. Gynecol. Oncol..

[20] Sun Y., Lu P., Yu L. (2016). The volume-metabolic combined parameters from 18F-FDG PET/CT may help predict the outcomes of cervical carcinoma. Acad. Radiol..

[21] Han S., Kim H., Kim Y.J., Suh C.H., Woo S. (2018). Prognostic value of volume-based metabolic parameters of 18F-FDG PET/CT in uterine cervical cancer: A systematic review and meta-analysis. AJR.

[22] Sharma D.N., Rath G.K., Kumar R., Malhotra A., Kumar S., Pandjatcharam J., Maharjan S. (2012). Positron emission tomography scan for predicting clinical outcome of patients with recurrent cervical carcinoma following readiation therapy. J. Cancer Res. Ther..

[23] Maharjan S., Sharma P., Patel C.D., Sharma D.N., Dhull V.S., Jain S.K., Thulkar S., Malhotra A., Kumar R. (2013). Prospective evaluation of qualitative and quantitative ^18^F-FDG PET-CT parameters for predicting survival in recurrent carcinoma of cervix. Nucl. Med. Commun..

[24] Anraku M., Yokoi K., Nakagawa K., Fujisawa T., Nakajima J., Akiyama H., Nishimura Y., Kobayashi K. (2004). Pulmonary metastases from uterine malignancies: Results of surgical resection in 133 patients. J. Thorac. Cardiovasc. Surg..

[25] Ki E.Y., Lee K.H., Park J.S., Hur S.Y. (2016). A clinicopathological review of pulmonary metastasis from uterine cervical cancer. Cancer Res. Treat..

[26] Oh J., Bae J.Y. (2018). Optimal cutoff level of serum squamous cell carcinoma antigen to detect recurrent cervical squamous cell carcinoma during post-treatment surveillance. Obstet. Gynecol. Sci..

[27] Duk J.M., van Voorst Vader P.C., ten Hoor K.A., Hollema H., Doeglas H.M., de Bruijn H.W. (1989). Elevated levels of squamous cell carcinoma antigen in patients with a benign disease of the skin. Cancer.

[28] Jao M.S., Chang T.C., Chang H.P., Wu T.I., Chao A., Lai C.H. (2010). Long-term follow up of cervical cancer patients with unexplained squamous cell carcinoma antigen elevation after post-therapy surveillance using positron emission tomography. J. Obstet. Gynaecol. Res..

[29] Sookha R.R., Zhi W., Shen Y., Lazare C., Wang L., Meng Y., Cao C., Hu J., Wu P. (2018). Clinical value of combining (18)f-fdg pet/ct and routine serum tumor markers in the early detection of recurrence among follow-up patients treated for cervical squamous cell carcinoma. J. Cancer.

[30] Salvatici M., Achilarre M.T., Sandri M.T., Boveri S., Vanna Z., Landoni F. (2016). Squamous cell carcinoma antigen (scc-ag) during follow-up of cervical cancer patients: Role in the early diagnosis of recurrence. Gynecol. Oncol..

[31] Hong J.H., Tsai C.S., Lai C.H., Chang T.C., Wang C.C., Chou H.H., Lee S.P., Hsueh S. (2004). Recurrent squamous cell carcinoma of cervix after definitive radiotherapy. Int. J. Radiat. Oncol. Biol. Phys..

[32] Liu Z., Shi H. (2019). Prognostic role of squamous cell carcinoma antigen in cervical cancer: A meta-analysis. Dis. Markers.

[33] Zhou Z., Liu X., Hu K., Zhang F. (2018). The clinical value of pet and pet/ct in the diagnosis and management of suspected cervical cancer recurrence. Nucl. Med. Commun..

[34] Ogino I., Nakayama H., Kitamura T., Okamoto N., Inoue T. (2005). The curative role of radiotherapy in patients with isolated para-aortic node recurrence from cervical cancer and value of squamous cell carcinoma antigen for early detection. Int. J. Gynecol. Cancer.

[35] Micco M., Varga H.A., Burger I.A., Kollmeier M.A., Goldman D.A., Park K.J., Abu-Rustum N.R., Hricak H., Sala E. (2014). Combined pre-treatment MRI and 18F-FDG PET/CT parameters as prognostic biomarkers in patients with cervical cancer. Eur. J. Radiol..

[36] Chung H.H., Kim J.W., Han K.H., Eo J.S., Kang K.W., Park N.H., Song Y.S., Chung J.K., Kang S.B. (2011). Prognostic value of metabolic tumor volume measured by FDG-PET/CT in patients with cervical cancer. Gynecol. Oncol..

[37] Rahman T., Tsujikawa T., Yamamoto M., Chino Y., Shinagawa A., Kurokawa T., Tsuchida T., Kimura H., Yoshida Y., Okazawa H. (2016). Different prognostic implications of ^18^F-FDG PET between histological subtypes in patients with cervical cancer. Medicine.

[38] Schoder H., Gonen M. (2007). Screening for cancer with pet and pet/ct: Potential and limitations. J. Nucl. Med..

[39] Chen Y.K., Ding H.J., Su C.T., Shen Y.Y., Chen L.K., Liao A.C., Hung T.Z., Hu F.L., Kao C.H. (2004). Application of pet and pet/ct imaging for cancer screening. Anticancer Res..

[40] Ide M. (2006). Cancer screening with fdg-pet. Q. J. Nucl. Med. Mol. Imaging.

